# Efficacy of the “Eiffel tower” double titanium elastic nailing in combined management of congenital pseudarthrosis of the tibia: preliminary outcomes of 17 cases with review of literature

**DOI:** 10.1186/s12891-021-04382-7

**Published:** 2021-05-28

**Authors:** Xiaoyu Wang, Li Shi, Rui Zhang, Wenbo Wang, Feng Wang, Mengwei Wang, Ze Xu, Rongtai Zuo, Jia Xu, Qinglin Kang

**Affiliations:** Department of Orthopaedic Surgery, Shanghai Jiao Tong University Affiliated Sixth People’s Hospital, 600 Yishan Road, Shanghai, 200233 China

**Keywords:** Congenital pseudarthrosis of the tibia (CPT), Intramedullary rod (IM rod), The “Eiffel tower” technique, Titanium elastic nail (TEN), Ilizarov technique

## Abstract

**Background:**

Difficulty in obtaining union, recurrent fractures, and residual deformities remain the problems challenging the management of congenital pseudarthrosis of the tibia (CPT). We applied the “Eiffel Tower” double titanium elastic nails (TENs) in the existing combined approach, which takes advantages of TEN’s mechanical stability with the protection against refracture, Ilizarov’s high fusion rate with alignment control and the biologic environment provided by bone grafting for bony union. The results of this procedure are presented and discussed.

**Methods:**

Seventeen patients with CPT treated by combined surgery including pseudarthrosis resection, the “Eiffel Tower” double TENs technique, autogenous iliac bone grafting, and Ilizarov fixation between 2013 and 2019 were retrospectively investigated. Signs of bone union, limb length discrepancy (LLD), rate of refracture, and degree of residual deformities were reviewed. The AOFAS Ankle Hindfoot scale and measurement of ankle motion were used to evaluate ankle function. The mean follow-up time was 40.5 (11 to 91) months.

**Results:**

The mean age at index surgery was 6.2 (2.5 to 15) years. Union of the pseudarthrosis was achieved in 100% of cases. Among them, 15 (88.2%) patients obtained union of the pseudarthrosis on the first attempt (primary union). The average time to primary union was 3.8 (2 to 6) months. The rest 2 cases achieved union after additional surgeries (secondary union). In terms of complications, refracture occurred in 2 patients (11.8%) and 4 patients (23.5%) developed pin infection. The mean limb length discrepancy at the final follow up was 33.4 (6–141) mm. The average AOFAS score improved from 38.2 (27 to 51) pre-operatively to 77 (63 to 87) post-operatively (*p* < 0.01).

**Conclusions:**

The “Eiffel Tower” double TENs technique is an ideal intramedullary fixation method in the surgical treatment of CPT. The combination of TENs technique with bone grafting and Ilizarov fixation has the advantages of early bone union, less injury on metaphysis, and early functional recovery.

**Level of evidence:**

Level IV.

## Introduction

Congenital pseudarthrosis of the tibia (CPT) is a rare and challenging orthopedic disease with over half of the patients associated with Neurofibromatosis Type 1 (NF-I) [1]. Currently, the combined treatment, including resection of the pseudarthrosis, autogenous bone grafting, and internal and external fixation, is the most commonly used technique. The combined surgical procedure takes the advantages of the external fixator’s high fusion rate with alignment control, protection against refracture provided by the internal fixation and the biologic environment facilitating bony union provided by bone grafting [2–5].

Rigid internal fixation is crucial to the treatment of CPT. The use of intramedullary (IM) rodding technique in treating CPT was first described by Charnley et al. in 1956 and was later modified by Williams et al. [6, 7]. The retrograde implanted transplantar intramedullary nails provide improved biomechanical stability for bone consolidation and restore the mechanical axis of the limb. Transfixion of the ankle and subtalar joints is usually required for providing enough stability to the pseudarthrosis site located at the distal tibia. However, joint stiffness and damage to the articular surface might occur in such situation. In addition, children usually have to receive reoperations as the tibiae outgrow their rods, which leads to interruption of childhood and prolonged disability [2, 8, 9]. Telescopic rods can extend during the growth and consequently decrease the number of reoperations. It should be noted, however, that the application of these rods in children is limited by the small shaft diameter of the medullary cavity. Moreover, iatrogenic injury may occur as the end of the telescopic rod still requires to be fixed through epiphysis [10].

The disadvantages of the commonly used IM rods encouraged us to make changes to the existing treatment procedure. Elastic stable intramedullary nails (ESINs, or Nancy nails) were initially developed in the 1980s by a group of pediatric surgeons in France [11]. The successful outcome of combining ESINs with external fixation for pediatric bone lengthening has led us to discover a new method of treating CPT [12]. During the past 10 years, the double titanium elastic nails (TENs) technique, in combination with pseudarthrosis resection, bone grafting, and Ilizarov’s fixation, has been applied to treat CPT patients in our institute. This study presents the outcomes of a retrospective case series that underwent this new fixation protocol and validates its efficacy by comparing with the results of previously used techniques.

## Materials and methods

We retrospectively evaluated the patients with pseudarthrosis of the tibia treated by the same group of senior surgeons at our institute from 2013 to 2019. Combined surgical treatment including pseudarthrosis resection, double TENs technique, autogenous iliac bone grafting, and Ilizarov fixation was set as inclusion criteria. Exclusion criteria included pseudarthrosis of the tibia caused by trauma or infection. A total of 17 patients (11 males and 6 females) with CPT were included. No patient was lost to follow-up. Background characteristics are shown in Table 1. Approval was obtained by the research ethics committee at Shanghai Jiao Tong University Affiliated Sixth People’s Hospital. All patients were informed consent to participate and approved the publication of their data.

**Table 1:** Demographic details of the patients

No	Sex	Previous surgery	Crawford type	Fibular lesion	Age at index surgery (yr)	Follow up period (mth)
1	F	Y	III	Y	2.9	91
2	M	N	IV	Y	5.3	74
3	M	Y	II	N	7.3	37
4	M	N	III	Y	2.9	40
5	F	Y	III	Y	12.3	28
6	F	Y	II	N	4.3	62
7	M	N	III	Y	5	31
8	M	Y	IV	Y	3.8	76
9	M	Y	III	Y	8.3	22
10	M	Y	IV	Y	15	37
11	M	Y	III	Y	11.1	32
12	M	N	III	Y	2.5	25
13	M	N	III	Y	4.2	39
14	F	N	IV	Y	5.7	31
15	F	N	III	Y	3.3	23
16	F	N	IV	N	3.4	11
17	M	Y	III	Y	8.2	30

*M* male, *F* female, *Y* yes, *N* no

### Pre-operative evaluation

Pre-operative measurements were performed, which included examination of skin condition, fibular status, and limb length discrepancy. Anteroposterior and lateral radiographs of the affected tibia were assessed using Crawford criteria. The length of the affected limb was measured on standing alignment radiographs and was compared with the reading of the contra-lateral leg pre-operatively. The American Orthopaedic Foot & Ankle Society [AOFAS] ankle-hindfoot scale was noted for clinical assessment of ankle function before the surgery [13]. All the cases were unilateral, with 7 cases on the right side and 10 cases on the left side. The pseudarthrosis were all located on the distal third of the tibia. Fourteen patients had a fibular lesion at the time of the index surgery. According to the Crawford classification, 0, 2, 10 and 5 patients were defined as type I, II, III and IV, respectively [14]. Mean age at index surgery was 6.2 (2.5 to 15) years and 9 patients had undergone surgery previously in other hospitals.

### Surgical procedure

**Fig. 1:**
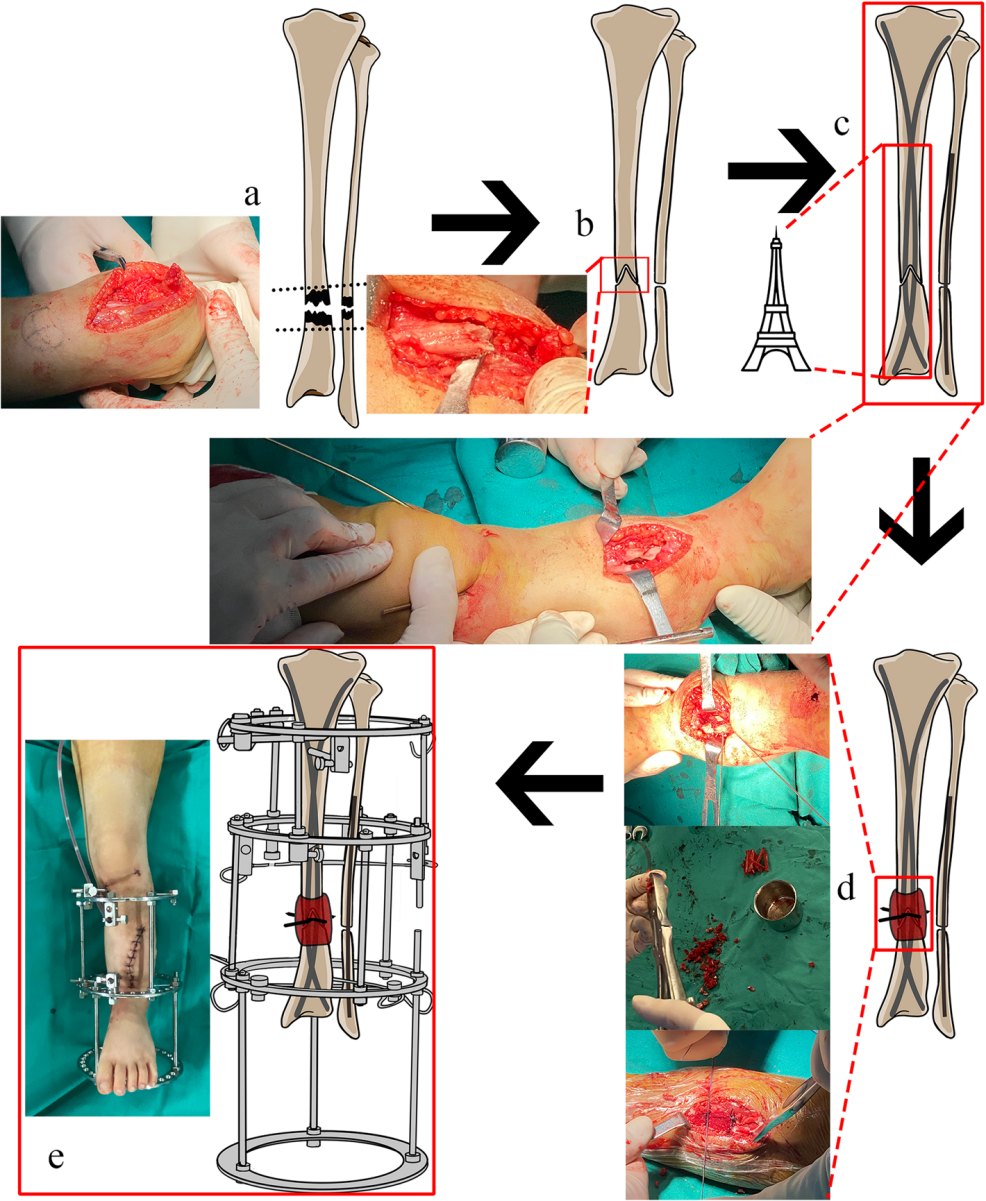
Schematic photographs and intra-operative appearance depicting the “Eiffel Tower” double titanium elastic nailing in combined management of CPT. Resection of the tibial pseudarthrosis (**a**). The proximal tibial stump was split into two flaps. The bone ends were docked (**b**) and two TENs were inserted into the tibia (**c**). Two additional k-wires were set in a crossed fashion for interlocking fixation at the pseudarthrosis site. The grafted cortex was cut into match-sticks’ size pieces, mixed with cancellous bone graft, and wrapped longitudinally around the docking area, secured with the absorbable sutures. The remaining cancellous bone graft compacted circumferentially between the grafted cortex pieces and the pseudarthrosis site (**d**). External fixator was applied (**e**). The drawn images were prepared by the authors

All surgeries were performed by the same senior surgeon (QL.K.) who has over fifteen years of experience in treating CPT. Schematic photographs and intra-operative appearance were shown in Fig. 1. At the beginning of the surgery, the outer table of the ilium was exposed subperiosteally and as much cancellous bone as possible was harvested from the supra-acetabular region for subsequent bone grafting. The exposed outer table of the ilium was cut into match-sticks’ size pieces for preparing grafted cortex. The abnormal periosteum and sclerotic bone edges at the pseudarthrosis site were excised. The fibrous tissue, if present at the fibular site in patients with concomitant fibular lesion, was excised. The fibula was osteotomized and fixed with an intramedullary k-wire if the tibial fragments were held apart by an intact fibula.

**Fig. 2:**
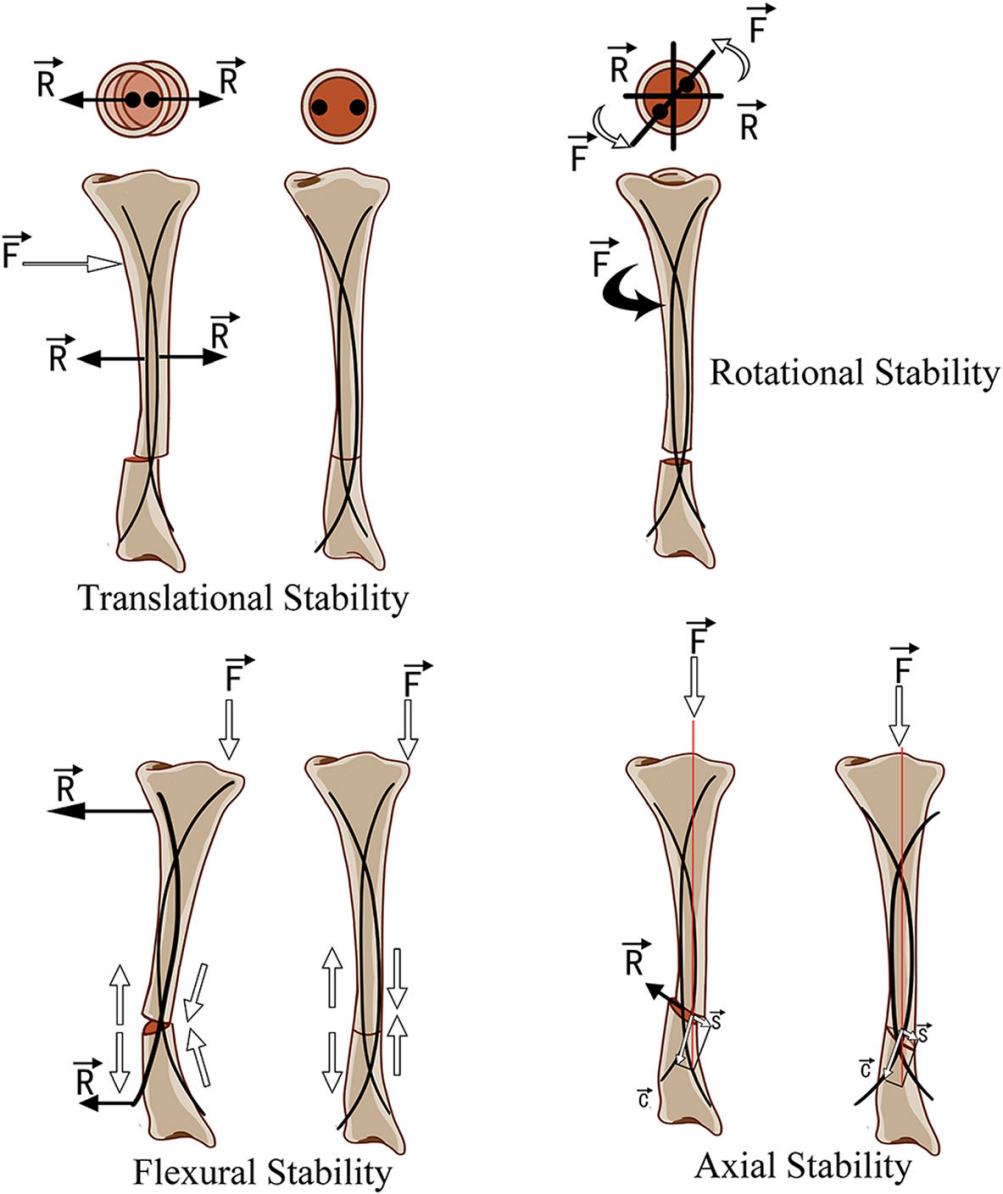
Biomechanical principles of double TENs in treating CPT. Two TENs were situated in a crossed fashion with their respective concavities facing each other. The translational, rotational, flexural and axial stability of the nails provide effective support for preventing further refracture and promoting solid union. F: Force acting on the bone; R: Restoring force of the nail; S: Shear force; C: Compressive force. The drawn images were prepared by the authors

Two TENs, with an identical diameter at 30–40% of the isthmus of the medullary canal (usually 1.5 to 2.5 cm), were selected for internal fixation. The entry points were situated anteriorly on the proximal medial and proximal lateral metaphyseal cortices, distal to the proximal physis. The first nail was inserted into the medullary canal with the nail tip at right angles to the bone shaft, and the nail tip was rotated through 180° with the inserter to align the nail tip with the axis of the medullary canal. Then the nail was advanced up across the resection site towards the level close to the distal tibia physis. The second nail was inserted at the insertion point on the opposite side in the same way. The two nails were situated in a crossed fashion like the contour of the Eiffel Tower. (Fig. 1c, Fig. 2).

The harvested cancellous grafts were placed around the site of pseudarthrosis of the tibia. The pieces of grafted cortex were wrapped longitudinally at the level of the pseudarthrosis area, secured with absorbable sutures. Cancellous bone grafts were filled circumferentially between the grafted cortex pieces and the pseudarthrosis site. Two additional k-wires (5–10 cm) were set in a cross fashion for interlocking fixation at the pseudarthrosis site. The Ilizarov ring fixation was then applied to perform realignment and compression osteosynthesis at the pseudarthrosis site. The wires of each ring should not be in contact with the TENs. A walking ring was added to eliminate the weight-bearing and LLD on the lower limb.

Active and passive motion of the joints were advised to prevent joint contracture after the surgery. Union was considered to be achieved with the formation of at least 3 visible bridging cortices in 2 planes under anteroposterior and lateral radiographs. Ilizarov frame was removed after bony union at the pseudarthrosis site. Long leg casting was then applied for 1–2 months. Hereafter patients were recommended moving under partial weight-bearing with the support of a Knee-Ankle-Foot-Orthosis until skeletal maturity. In order to minimize the risk of refracture and abnormal tibia bowing, the TENs were left in situ till skeletal maturity but may be changed for longer and thicker ones when the TENs appear undersized with growth.

### Outcome evaluation

Post-operative radiographs were obtained every month until fixator removal. Thereafter, follow-ups were continued every half year until the patients achieved skeletal maturity. Primary union referred to bone united after the first operation without secondary surgeries. Secondary union was labeled when additional surgery was needed to obtain union. Limb length was measured radiographically from proximal physis to distal physis of the tibia. It should be mentioned that LLD measurement was performed soon after the index surgery as LLD might increase due to impaction of bone ends after excision of pseudarthrosis. Residual deformities, including tibial malalignment and ankle valgus, were assessed by the anteroposterior and lateral radiographs. To assess the ankle function after the surgery, a goniometer was used to measure the range of motion (ROM). The AOFAS scale was used to make comprehensive evaluation of the ankle function post-operatively [13].

### Statistical methods

Descriptive statistics were used for analyzing radiographic measurements and demographic characteristics. All statistics were calculated using IBM SPSS software (version 22). Paired t-test was used for evaluating the difference between pre- and post-operative AOFAS score (at last follow-up). The paired Wilcoxon test was performed for analysis of LLD post-operation and at the final follow up. The level of significance was set at *p* < 0.01.

## Results

**Fig. 3:**
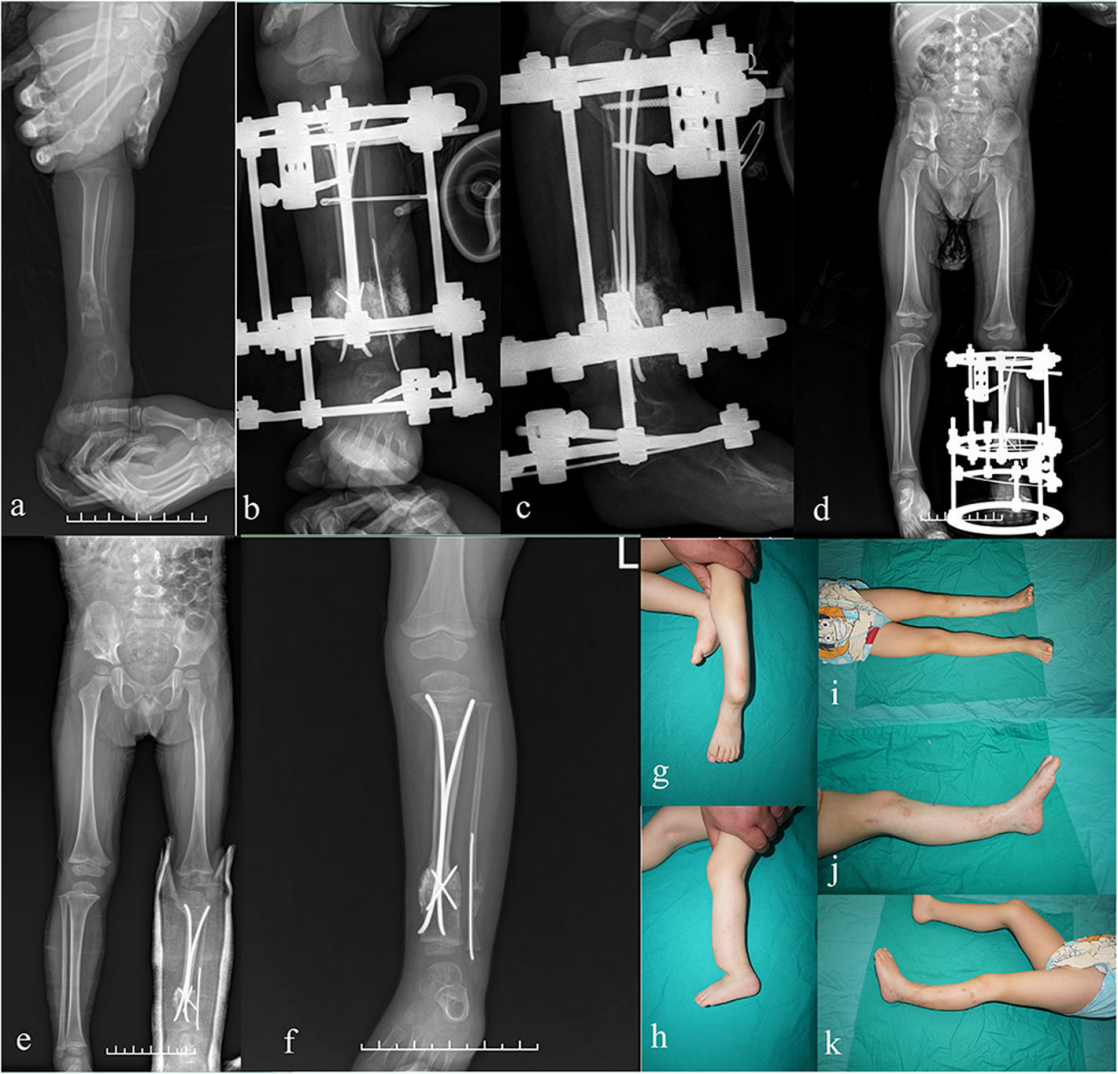
Case 12, a 2.5-year-old boy with CPT treated successfully with pseudarthrosis resection, the “Eiffel Tower” double TENs, bone grafting and external fixation. Pre-operative anteroposterior X-rays showing Crawford type III CPT with anterolateral bowing of tibia (**a**). Anteroposterior and lateral radiograph of the same patient taken immediately after combined surgery (**b**, **c**). X-ray taken 3 months post-operatively shows primary union of the pseudarthrosis site (**d**). Radiograph taken 6 months post-operatively shows a well-aligned and remodeled tibia without ankle valgus or tibia angulation (**e**). The nails were retained in the tibia medullary cavity with growth (**f**). Clinical appearance of the patient before (**g**, **h**) and after (**i**, **j**, **k**) the surgery. The patient had an AOFAS score of 82 and a 20 mm leg-length shortening at the finally follow-up

**Fig. 4:**
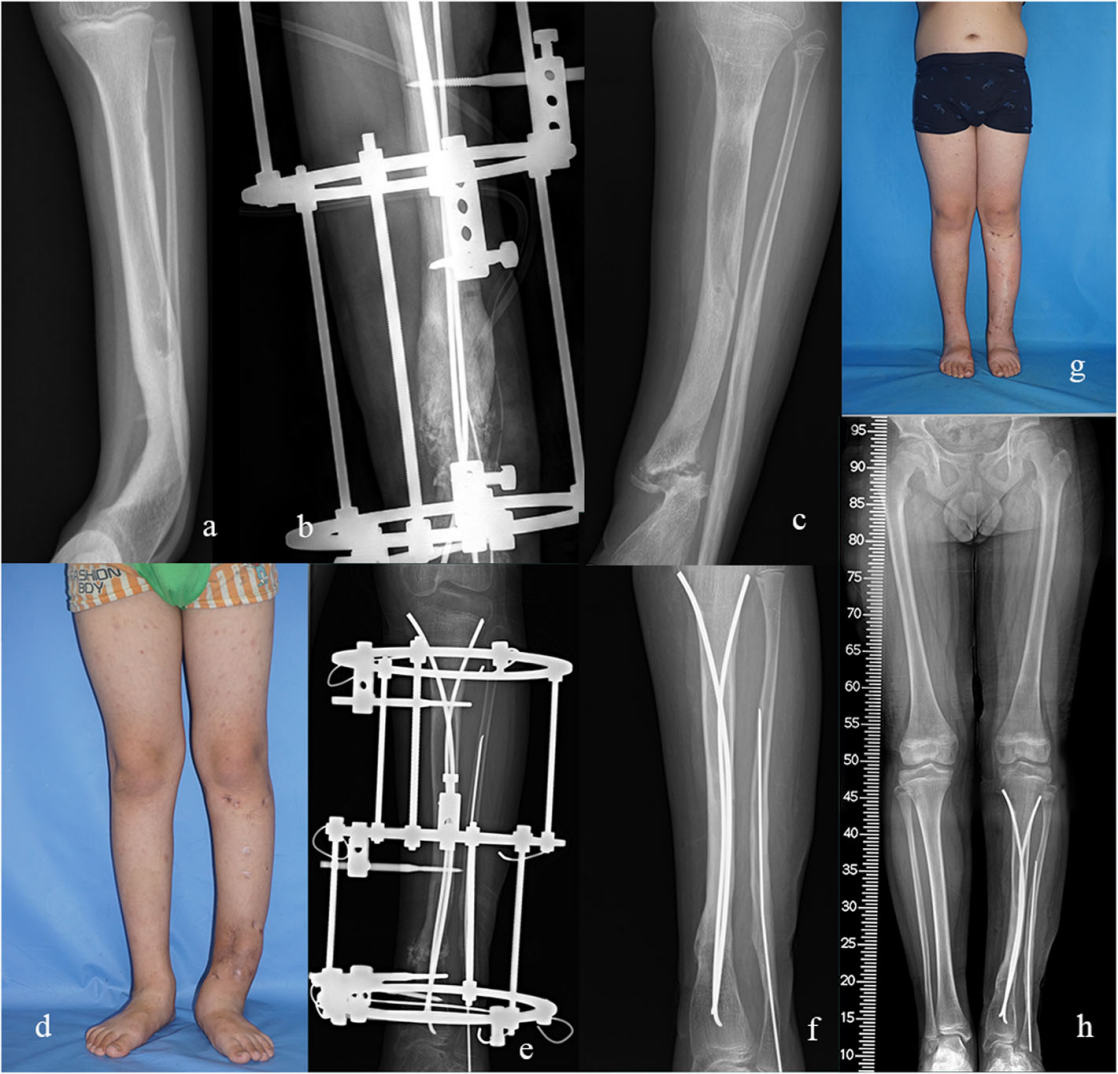
Anteroposterior radiograph of a 7.3-year-old boy with Crawford type II CPT (Case 3) (**a**). He underwent additional bone grafting and prolonged external fixation due to delayed union after initial combined surgery (**b**). However, refracture occurred 1 month after removal of Ilizarov fixator. He received cast immobilization and removal of broken nails at local medical center (**c**, **d**). In our institute, double TENs insertion and external fixation were applied (**e**). The fracture healed 5 months later and the external fixator was removed (**f**). Clinical and radiological appearance shows a well-aligned lower limb and sound union of the pseudarthrosis site (**g**, **h**)

The external frames were removed, and the TENs were retained in all of the 17 patients at the latest follow-up (Fig. 3). Refracture occurred in 2 patients (11.8%, case 3 and 6), both after removal of the external fixators (1 month and 4 years after removal of the external fixation). Case 3 underwent bone graft and prolonged external fixation due to delayed union. Unfortunately, refracture occurred due to the unwillingness of brace wearing. He received removal of the broken nails and cast immobilization in the local medical center. TENs reinsertion and external fixation were then applied in our institute (Fig. 4). In case 6, the patients received fracture reduction and nails reinsertion (Table 2).

**Table 2:** Bone union, refracture, length discrepancy and complications of the patients

No	Union time (mth)	Number of operations before union	Type of union	Po-op LLD (mm)	LLD at final follow-up (mm)	Complications	Additional Treatment
1	5	1	Primary	42	6	Left ankle valgus	Hemiepiphyseodesis of medial distal tibia
							Limb lengthening
2	4	1	Primary	31	12	Right ankle valgus	Hemiepiphyseodesis of medial distal tibia
3	10	3	Secondary	50	23	Pin tract infection	Dressing change and oral administration of antibiotics
						Delayed union	Prolonged external fixation, bone graft
						Refracture (1 month after	Cast immobolization, then nail reinsertion
						external fixation removal)	and external fixation
4	3	1	Primary	27	17	None	None
5	5	1	Primary	63	42	None	None
6	6	1	Primary	32	24	Tibia valgus and recurvature	Nail change
						Refracture (4 years after initial union)	Hemiepiphyseodesis of medial distal tibia, fracture reduction, nails reinsertion
7	6	1	Primary	60	41	None	None
8	2	1	Primary	47	30	Right ankle valgus	Hemiepiphysiodesis of medial distal tibia
9	3	1	Primary	40	22	Left knee valgus	Hemiepiphysiodesis of medial proximal tibia
10	11	2	Secondary	135	141	Pin tract infection	Dressing change and oral administration of antibiotics
						Delayed union	Debridement, nail change, drainage of wound, prolonged external fixation and bone graft
							Limb lengthening
11	6	1	Primary	52	35	Pin tract infection	Dressing change and oral administration of antibiotics
12	3	1	Primary	13	20	None	None
13	4	1	Primary	55	29	Pin tract infection	Dressing change and oral administration of antibiotics
14	6	1	Primary	64	48	None	None
15	3	1	Primary	45	20	None	None
16	4	1	Primary	43	22	None	None
17	5	1	Primary	38	35	None	None

*Po-op* post-operative, *LLD* limb length discrepancy

Union of the pseudarthrosis was achieved in 100% of the cases, with primary union in 15 cases (88.2%). Two cases of delayed union at the pseudarthrosis site (case 3 and case 10) achieved union after prolonged external fixation and additional bone graft. (Table 2). The average time to primary union was 3.8 (2 to 6) months and for secondary union it was 10.5 (10 to 11) months. On average, 1.2 (1 to 2) surgeries were performed to achieve union (Table 2).

The average limb length discrepancy after index surgery was 49.2 (13 to 135) mm. The mean limb-length discrepancy at the final follow-up was 33.4 (6 to 141) mm (*p* < 0.01). Five patients developed residual deformities and require further operations (Table 3). Among them, three patients (case 1, 2 and 8) developed valgus deformity of the ankle, with lateral distal tibial angle (LDTA) of 74°, 68° and 81°, respectively. Hemiepiphyseodesis of medial distal tibia was applied in all 3 patients. One patient (case 9) developed knee valgus, with medial proximal tibial angle (MPTA) of 96°. Hemiepiphysiodesis of medial proximal tibia was applied for guiding the growth towards the normal alignment. One patient (case 6) developed tibia valgus and received additional surgery for nail change. As shown in Table 3, the mean pretreatment AOFAS score for all 17 patients was 38.2 (27 to 51), which increased significantly to 77 (63 to 87) at the time of the last follow-up (*p* < 0.01). Tibiotalar joint was free in 15 (88.2%) patients at the final follow up, with an average range of movement of 23 (15-30) °during dorsiflexion and 37 (30–40) ° during plantar flexion.

**Table 3:** Pre- and post-operative ankle function of the patients

No	Residual angular deformity	Pre-op AOFAS score	AOFAS score at last follow-up	Tibiotalar range of motion (°) (dorsiflexion/plantarflexion)	Subtalar range of motion (°) (eversion/inversion)
1	Valgus deformity of left ankle (LDTA: 74°)	39	84	20/40	10/15
2	Valgus deformity of right ankle (LDTA: 68°)	42	79	20/40	10/20
3	None	27	70	10/15	10/10
4	None	51	80	25/40	10/25
5	None	33	73	25/40	15/25
6	None	47	78	30/35	20/20
7	None	49	87	20/40	10/20
8	Valgus deformity of right ankle (LDTA: 81°)	48	72	25/40	15/25
9	Valgus deformity of left knee (MPTA: 96°)	43	63	5/10	5/0
10	None	39	76	15/30	10/15
11	None	38	73	25/30	10/25
12	None	28	82	20/40	15/20
13	None	27	78	30/40	15/20
14	None	39	82	25/35	10/20
15	None	36	79	20/35	15/15
16	None	27	73	20/40	20/20
17	None	36	80	25/30	15/15

*Pre-op* pre-operative

Pin tract infection was identified in 4 patients, all were settled with dressing changes. One patient (case 10) received additional debridement, nail change and drainage of wound. There were no cases of neurovascular complications or amputation in our study.

## Discussion

The achievement of skeletal maturity with functional utility and anatomic alignment is the primary goal of treating CPT [15, 16]. Due to the high rate of refracture, pediatric surgeons were compelled to focus on successful osteosynthesis rather than the sequelae of CPT, such as joint stiffness and arthritic change led by trans-ankle fixation; metaphyseal irritation and growth failure led by metaphyseal intrusion; and even failure of IM fixation due to inadequate space for nail interlocking in pediatric patients [2, 8, 10, 17–19]. The disadvantages of conventional IM rods encouraged us to make changes to the treatment procedure with technical modifications. The combination of IM rodding, external fixation, and iliac bone grafting have been generally accepted as an effective procedure in protecting against refracture, promoting alignment control with high fusion rate and contributing to early removal of external fixator [2–5, 20]. Since 2013, we applied the “Eiffel Tower” double TENs instead of conventional IM rods for internal fixation. The two TENs were positioned in crossed fashion (the “Eiffel Tower” method) in the medullary canal of the affected tibia, with their respective concavities facing each other (Fig. 1c).

Our study has shown favorable results, with primary union obtained in 88.2% of patients. An average number of 1.2 (1 to 2) surgeries was performed to achieve solid union for each patient, which is in line with that reported previously [5]. Refracture occurred in only 2 (11.8%) of 17 patients. All obtained union after additional surgery. In one patient (case 6), the occurrence of refracture was probably because of persistent valgus deformity and recurvature of the tibia, although nail change was performed. She later received hemiepiphyseodesis of medial distal tibia for guiding the growth of tibia. Another patient (case 3) experienced pin tract infection and delayed consolidation, and eventually obtained secondary union. Unfortunately, he failed to keep brace wearing and refracture occurred after trauma. These results were similar or even superior to other studies [16]. Dobbs et al. reported that trans-ankle Williams rod showed satisfactory long-term outcomes, with initial consolidation obtained in 18 (85.7%) of 21 patients and 12 (23.2%) cases of refracture. They considered that removing the rod after union for regaining ankle motion was inadvisable due to the high risk of refracture. They also noted that the frequency of refracture was higher when fibular pseudarthrosis was not treated [9]. In a series of 15 cases that received IM nailing combined with Ilizarov fixation, Agashe et al. reported that 14 patients (93.3%) achieved union, with primary union in 6 patients. During the 4.2-year follow-up period, only 1 patient (6.7%) developed refracture. They further concluded that undue stress led by the persistent malalignment of the tibia and fibula, loss of intramedullary fixation, and non-compliance with external bracing regimen are three major causes of refracture [2]. Refracture occurred in 13 (23.2%) of 56 patients treated with combined surgery including pseudarthrosis resection, intramedullary Williams rodding, autogenous iliac bone grafting, and Ilizarov’s fixator in the Zhu et al. study [3].

The elastic deformation of the TENs creates a bending moment within the long bone that is not rigid but stable enough to provide effective support for preventing further refracture and promoting solid union. As shown in Fig. 2, the Eiffel Tower shaped elastic internal frame with six-point fixation ensures flexural, translational, rotational, and axial stability of the pseudarthrosis site under external forces. The enhanced bone formation along the tract of TENs, probably due to friction or stimulatory irritation within the intramedullary canal, and a preserved intramedullary blood supply, are considered to be another underlying explanation for earlier union and lower rate of refracture, as described by Popkov et al. [12].

Paley defined the success probability as:

*Success probability = Union rate × [1 − Mean refracture rate].*

He reported that on average, success probability was 40% in intramedullary rodding, 57% in the Ilizarov method, and 58% in intramedullary rodding combined with the Ilizarov method [21]. In our study, the success probability was 77.9%. However, it should be noted that additional refractures may occur before patients reach skeletal maturity. Thus, final outcomes should be assessed after all the patients have reached this milestone.

With longitudinal growth of the lower extremity, the distal part of the tibia, the ankle, and the foot migrate distally while the rod remains in place. Thus, reoperations are usually required to push the rod across the ankle joint for continuous fixation till skeletal maturity [2, 9, 10]. Telescopic rods, such as Bailey-Dubow rods, can elongate as the child grows, which helps to decrease the number of reoperations required for these children. However, disengagement of the epiphyseal T-piece remains the major pitfall [19]. The later technically modified expandable rods provided by the Sheffield group ensured the better fixation within the epiphysis but knee and ankle arthrotomies are still required for a tibial insertion of 2 telescoping components [22]. The subsequently developed Fassier-Duval Rod managed to further decrease the reoperation rate by making the insertion with a single proximal entry. Unfortunately, due to the lack of longitudinal and rotational stability, the sole use of Fassier-Duval rod fixation in patients with severe underlying bone pathology of CPT end up with discouraging nonunion, collapse (“negative telescoping”) and consecutive joint intrusion [10, 23]. The high cost also limits its wide acceptance in the developing area. It is also technically difficult to apply these rods in bones with severe curvature or multiple shaft deformities [12]. On the contrary, the double TENs technique did not require joint transfixation or arthrotomies. Thus, nail changing for a larger one was not performed frequently, unless tibia curvature or an obvious mismatch between the length of the TENs and tibia occurs. The entry points for pulling out and inserting TENs were set distal to the proximal physis, which makes the procedure feasible and less invasive.

Three of 17 patients (17.6%) developed ankle valgus and all of them had received surgeries on fibular lesions. There seems to be a trend toward an increased rate of post-operative ankle valgus in the patients with fibular pseudarthrosis, probably due to a high position of the fibular distal epiphysis and an asymmetric growth of the distal tibial physis that grows more medially than laterally [20, 24]. Moreover, proximal migration of the distal fibula causes the talus to move follow the fibula, which contributes to ankle valgus and lateral subluxation of the ankle joint. Ankle deformities have been reported to develop over time in the patients with pseudarthrosis remains un-united. The resultant instability of the ankle increases the risk of refracture [2, 9, 21, 25]. Thus, the sooner the integrity of the fibula is achieved, the better the chances of avoiding them. Distal tibiofibular synostosis recommended by Thabet et al. is useful in fibular healing, deformity control and refracture treatment [26]. Hemi-epiphysiodesis is the most favorable method to correct ankle valgus in CPT children with enough growth potential, as it is less invasive and can avoid the risk of nonunion brought by corrective osteotomy. In our study, all three patients obtained restored ankle joint alignment asymptomatically at the last follow-up.

Karol et al. demonstrated that patients with trans-articular intramedullary nails developed 68% diminished ankle push off strength while such value was only 36% in the group that did not have the rod inserted across the joint [27]. Retaining the trans-articular nail for a long period of time may lead to ankle stiffness and gait abnormalities [2, 8, 19]. Dobbs et al. used the solid two-part Williams intramedullary rod and recommended surgically advancing the rod out of the ankle joint soon after the pseudarthrosis has healed [9]. However, metaphyseal irritation and arthritic change of ankle joint remained as the rod is advanced antegrade across the ankle joint and the ankle joint is usually immobilized by rod transfixation for over 2 years after rod insertion [8, 9, 18, 24]. Custom interlocking intramedullary nails may decrease the prevalence of ankle stiffness as it does not transfix the ankle joint. For younger patients and patients with relatively distal location of the pseudarthrosis the tibia, bone segment distal to the pseudarthrosis is not long enough for adequate interlocking fixation [24]. In our study, the ends of the nails were at the level of 1 cm distal to the metaphysis of tibia, which protects the ankle joints from being jeopardized by classical rodding through the ankle. In addition, early ankle function recovery by active and passive motion is also available. Range of motion in tibiotalar and subtalar joints was optimal in 15 (88.2%) patients. Only 2 patients in our study developed subtalar and tibiotalar stiffness, probably due to relatively distal location of pseudarthrosis (case 3) and retrograde intramedullary rodding in the previous failed surgery (case 9).

Agashe et al. reported that under the treatment of Ilizarov technique combined with intramedullary rodding, the mean AOFAS score increased from 40 to 64 during a mean follow-up time of 4.5 years [2]. Our study presents relatively favorable results, with the average AOFAS increased from 38.2 to 77 (*p* < 0.01). Although a relatively longer follow up period in our study (3.4 years) may lead to a slightly higher AOFAS score at the final follow up, it is still obvious that the ankle function improved greatly during the follow-up period.

Length discrepancy is a common challenge resulting from inhibited growth of distal physis, surgical resection, and bone resorption at pseudarthrosis [21]. It should be noted that proximal tibia dysplasia and repeated lengthening were identified as the risk factors of poor regenerate bone formation at the distraction site in CPT patients [28, 29]. On the other hand, one stage lengthening may affect the healing process at the pseudarthrosis site. Postponing tibial lengthening after initial union was considered to have fewer side effect on the pseudarthrosis but may cause larger LLD and a prolonged period of external fixation [29]. Previous study has reported the successful outcome of combining TENs with external fixation for pediatric bone lengthening [12]. We are proceeding with a long-term study to find out the exact time window for proximal tibia lengthening with double TENs technique which will yield the most favorable outcomes.

The small number of patients is the limitation of this study. Due to the retrospective nature of the study and the heterogeneous treatment for each patient, the efficacy of double TENs technique with other approaches combined, as well as bone transportation, still cannot be confirmed directly from our study. The true success of treating CPT in a growing child can be confirmed only by following the children until maturity. In all, a well-designed prospective study with larger numbers of patients included is required.

In conclusion, this study demonstrated that the application of double titanium elastic nails (TENs) with the “Eiffel Tower” technique, combined with bone grafting and Ilizarov method, is a viable option for CPT in achieving and maintaining early union. The advantages lie in its good stability and protection against refracture. The readily availability is another factor that makes it an alternative surgical option for young children in the developing world. Moreover, compared with other rodding techniques, the TENs technique poses less injury to ankle joint and metaphysis, which effectively avoids ankle stiffness and reduces negative impact on tibia growth.

## Data Availability

The datasets in the current study are available from the corresponding author on reasonable request.

## References

[1] Hefti F, Bollini G, Dungl P, Fixsen J, Grill F, Ippolito E (2000). Congenital pseudarthrosis of the tibia: history, etiology, classification, and epidemiologic data. J Pediatr Orthop B.

[2] Agashe MV, Song SH, Refai MA, Park KW, Song HR (2012). Congenital pseudarthrosis of the tibia treated with a combination of Ilizarov's technique and intramedullary rodding. Acta Orthop.

[3] Zhu GH, Mei HB, He RG, Liu YX, Liu K, Tang J (2016). Combination of intramedullary rod, wrapping bone grafting and Ilizarov's fixator for the treatment of Crawford type IV congenital pseudarthrosis of the tibia: mid-term follow up of 56 cases. BMC Musculoskelet Disord.

[4] Kocaoğlu M, Eralp L, Bilen FE, Civan M (2020). Congenital pseudarthrosis of the tibia: results of circular external fixation treatment with intramedullary rodding and periosteal grafting technique. Acta Orthop Traumatol Turc.

[5] Shabtai L, Ezra E, Wientroub S, Segev E (2015). Congenital tibial pseudarthrosis, changes in treatment protocol. J Pediatr Orthop B.

[6] Charnley J (1956). Congenital pseudarthrosis of the tibia treated by intramedullary nail. J Bone Joint Surg Am.

[7] Williams PF (1965). Fragmentation and rodding inOsteogenesis imperfecta. J Bone Joint Surg Bri volume.

[8] Shah H, Doddabasappa SN, Joseph B (2011). Congenital pseudarthrosis of the tibia treated with intramedullary rodding and cortical bone grafting: a follow-up study at skeletal maturity. J Pediatr Orthop.

[9] Dobbs MB, Rich MM, Gordon JE, Szymanski DA, Schoenecker PL (2004). Use of an intramedullary rod for treatment of congenital pseudarthrosis of the tibia. A long-term follow-up study. J Bone Joint Surg Am.

[10] Birke O, Davies N, Latimer M, Little DG, Bellemore M (2011). Experience with the Fassier-Duval telescopic rod: first 24 consecutive cases with a minimum of 1-year follow-up. J Pediatr Orthop.

[11] Ligier JN, Metaizeau JP, Prévot J, Lascombes P (1988). Elastic stable intramedullary nailing of femoral shaft fractures in children. J Bone Joint Surg Bri volume.

[12] Popkov A, Foster P, Gubin A, Borzunov D, Popkov D (2017). The use of flexible intramedullary nails in limb lengthening. Expert Rev Med Devices.

[13] Kitaoka HB, Alexander IJ, Adelaar RS, Nunley JA, Myerson MS, Sanders M (1994). Clinical rating systems for the ankle-hindfoot, midfoot, hallux, and lesser toes. Foot Ankle Int.

[14] Crawford AH, Schorry EK (1999). Neurofibromatosis in children: the role of the orthopaedist. J Am Acad Orthopaedic Surgeons.

[15] O'Donnell C, Foster J, Mooney R, Beebe C, Donaldson N, Heare T. Congenital Pseudarthrosis of the Tibia. JBJS reviews. 2017;5(4):e3. 10.2106/JBJS.RVW.16.00068 28437289

[16] Kesireddy N, Kheireldin RK, Lu A, Cooper J, Liu J, Ebraheim NA (2018). Current treatment of congenital pseudarthrosis of the tibia: a systematic review and meta-analysis. J Pediatr Orthop B.

[17] Chalopin A, Pesenti S, Peltier E, Bin K, Launay F, Jouve JL (2016). Transplantar intramedullary locking nailing in childhood congenital pseudarthrosis of the tibia: a report of 3 cases. Orthopaedics Traumatol Surg Res: OTSR.

[18] Seo SG, Lee DY, Kim YS, Yoo WJ, Cho TJ, Choi IH (2016). Foot and ankle function at maturity after Ilizarov treatment for atrophic-type congenital Pseudarthrosis of the tibia: a comprehensive outcome comparison with Normal controls. J Bone Joint Surg Am.

[19] Gamble JG, Strudwick WJ, Rinsky LA, Bleck EE (1988). Complications of intramedullary rods in osteogenesis imperfecta: bailey-Dubow rods versus nonelongating rods. J Pediatr Orthop.

[20] Mathieu L, Vialle R, Thevenin-Lemoine C, Mary P, Damsin JP (2008). Association of Ilizarov's technique and intramedullary rodding in the treatment of congenital pseudarthrosis of the tibia. J Child Orthop.

[21] Paley D (2019). Congenital pseudarthrosis of the tibia: biological and biomechanical considerations to achieve union and prevent refracture. J Child Orthop.

[22] Wilkinson JM, Scott BW, Clarke AM, Bell MJ (1998). Surgical stabilisation of the lower limb in osteogenesis imperfecta using the Sheffield telescopic intramedullary rod system. J Bone Joint Surg Bri volume.

[23] Cho TJ, Choi IH, Chung CY, Yoo WJ, Lee KS, Lee DY (2007). Interlocking telescopic rod for patients with osteogenesis imperfecta. J Bone Joint Surg Am.

[24] Johnston CE (2002). Congenital pseudarthrosis of the tibia: results of technical variations in the charnley-Williams procedure. J Bone Joint Surg Am.

[25] Choi IH, Lee SJ, Moon HJ, Cho TJ, Yoo WJ, Chung CY (2011). "4-in-1 osteosynthesis" for atrophic-type congenital pseudarthrosis of the tibia. J Pediatr Orthop.

[26] Thabet AM, Paley D, Kocaoglu M, Eralp L, Herzenberg JE, Ergin ON (2008). Periosteal grafting for congenital pseudarthrosis of the tibia: a preliminary report. Clin Orthop Relat Res.

[27] Karol LA, Haideri NF, Halliday SE, Smitherman TB, Johnston CE (1998). 2nd. Gait analysis and muscle strength in children with congenital pseudarthrosis of the tibia: the effect of treatment. J Pediatr Orthop.

[28] Cho TJ, Choi IH, Lee KS, Lee SM, Chung CY, Yoo WJ (2007). Proximal tibial lengthening by distraction osteogenesis in congenital pseudarthrosis of the tibia. J Pediatr Orthop.

[29] Zhu GH, Mei HB, He RG, Liu K, Tang J, Wu JY (2015). Effect of distraction osteogenesis in patient with tibial shortening after initial union of congenital Pseudarthrosis of the tibia (CPT): a preliminary study. BMC Musculoskelet Disord.

